# Techniques, Databases and Software Used for Studying Polar Metabolites and Lipids of Gastrointestinal Parasites

**DOI:** 10.3390/ani14182671

**Published:** 2024-09-13

**Authors:** Phurpa Wangchuk, Karma Yeshi

**Affiliations:** 1 College of Public Health, Medical and Veterinary Sciences (CPHMVS), James Cook University, McGregor Rd, Smithfield, Cairns, QLD 4878, Australia; phurpa.wangchuk@jcu.edu.au; 2 Australian Institute of Tropical Health and Medicine (AITHM), James Cook University, McGregor Rd, Smithfield, Cairns, QLD 4878, Australia

**Keywords:** gastrointestinal parasites, small molecules, database, software, metabolomics, lipidomics, artificial intelligence

## Abstract

**Simple Summary:**

Gastrointestinal parasites (GIPs) coevolved with mammalian hosts over millennia. These parasites produce small molecules, peptides, and proteins not only to evade or combat the host’s immune response, but also to protect their host for longer coexistence. The emerging field of parasitomics uses various techniques, databases, and software associated with LC-MS (liquid chromatography–mass spectrometry), NMR (nuclear magnetic resonance), and other mass spectrometry platforms to study the polar and lipid molecules produced by GIPs. Recent advancements in AI-assisted tools and databases have significantly advanced this field, offering new insights into host–parasite interactions, immunomodulation and biochemical pathways. As research progresses, parasitomics promises to deepen our understanding of these complex relationships.

**Abstract:**

Gastrointestinal parasites (GIPs) are organisms known to have coevolved for millennia with their mammalian hosts. These parasites produce small molecules, peptides, and proteins to evade or fight their hosts’ immune systems and also to protect their host for their own survival/coexistence. The small molecules include polar compounds, amino acids, lipids, and carbohydrates. Metabolomics and lipidomics are emerging fields of research that have recently been applied to study helminth infections, host–parasite interactions and biochemicals of GIPs. This review comprehensively discusses metabolomics and lipidomics studies of the small molecules of GIPs, providing insights into the available tools and techniques, databases, and analytical software. Most metabolomics and lipidomics investigations employed LC-MS, MS or MS/MS, NMR, or a combination thereof. Recent advancements in artificial intelligence (AI)-assisted software tools and databases have propelled parasitomics forward, offering new avenues to explore host–parasite interactions, immunomodulation, and the intricacies of parasitism. As our understanding of AI technologies and their utilisation continue to expand, it promises to unveil novel perspectives and enrich the knowledge of these complex host–parasite relationships.

## 1. Introduction

Metabolomics and lipidomics are powerful techniques developed to study polar metabolites and lipids (both endogenous and exogenous) present in biological samples or organisms [1]. These techniques help us to understand the genotype or phenotype of the biological system at the biochemical level, which has immense applications in health and disease research [2]. These platforms mainly rely on NMR (nuclear magnetic resonance) spectroscopy and mass spectrometry, including gas chromatography–mass spectrometry (GC-MS), liquid chromatography–mass spectrometry (LC-MS), and capillary electrophoresis–mass spectrometry (CE-MS) [2,3]. Compared to conventional natural products isolation and structure elucidation methods, all these techniques are high-throughput systems that can measure and identify many metabolites at a time.

Metabolomics and lipidomics are relatively new “omics” approaches applied in helminth research, with immense potential for identifying metabolites in cells, biofluids (including excretory/secretory products, ESPs), tissues, and whole organisms [4]. Gastrointestinal parasites (GIPs) have coevolved with their hosts for millennia [5]. The host and the parasite compete for available energy resources and metabolic building blocks while parasites enter the gut, significantly impacting each other’s metabolic homeostasis [4,6]. Historically, parasitic helminths’ biological and molecular complexities have posed challenges in understanding the biochemicals and their biological functions. However, the emergence of “omics” technologies has enabled a deeper understanding of parasites at a systems biology level, which is crucial for advancements in preventing and treating helminth infections [7].

Different types of metabolomics and lipidomics workflows have been applied based on the choice of experimental approach (targeted versus untargeted approach) [8]. Unlike the targeted approach, the untargeted approach generates more complex data, the processing and interpretation of which require advanced computational methods, such as AI (artificial intelligence) and ML (machine learning) algorithms. Advanced AI platforms are crucial tools in processing and analysing complex untargeted data [9,10], as they can eliminate background noise in peak identification, alignment, and normalisation [10]. Typically, ML algorithms are used to build mathematical models from a data set in a series of steps [10,11]. They have offered countless opportunities, including early disease diagnosis [12], reviewed in depth by Galal et al. [10].

In this review, we retrieved published metabolomics and lipidomics studies on small molecules from various gastrointestinal parasites, sourcing data from databases such as PubMed, MEDLINE Ovid, Scopus, Google Scholar, and Web of Science. To retrieve relevant literature, we used the following keywords: “metabolomics techniques”, “lipidomics”, “targeted metabolomics”, “untargeted metabolomics”, “small molecules”, “helminths-derived small molecules”, “host-parasite communication”, “metabolites”, “lipids”, and “excretory and secretory products” and gastrointestinal parasite names. We summarised the AI-assisted metabolomics and lipidomics techniques, databases, and analytical software used for studying various gastrointestinal parasites.

## 2. Interaction of Gastrointestinal Parasite with Animal Hosts Using Small Molecules

**Figure 1:**
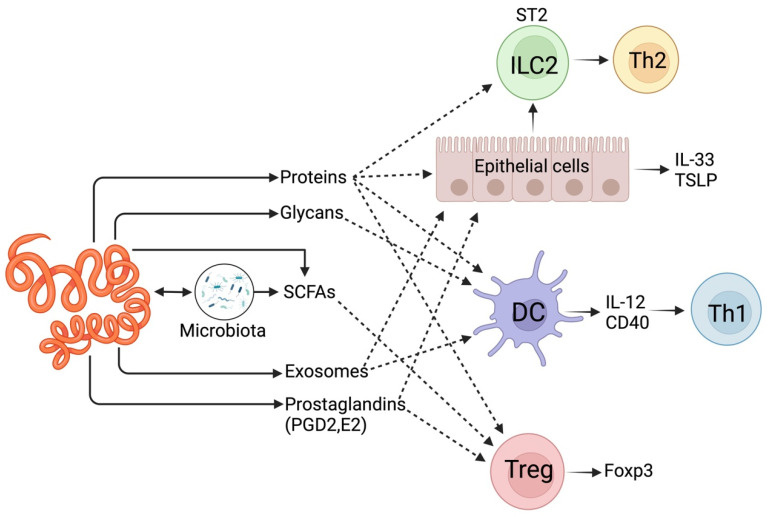
Host–pathogen recognition systems during GI helminth infections. The host innate immune system responds to injury by releasing alarmins (e.g., IL-33, TSLP), initiating a type 2 (Th2) immune response. However, GI helminths may block the release of alarmins or their receptors, e.g., IL-33R (or ST2). Toll-like receptors (TLRs) may recognise pathogen-associated molecular patterns (PAMPs). These PAMPs can be presented directly by helminths or indirectly by bacteria passing through the injured epithelium. In such cases, helminths secrete immune modulators that block the Th1 response driven by IL-12.

Gastrointestinal parasites interact with their host in different ways to survive inside the host or to complete their life cycle [13]. Adult parasites, as well as those in the developmental cycle, excrete/secrete (ES) various small molecules and metabolites to manipulate the host’s immune system [14]. There is growing interest in how these small molecules and other metabolites and molecular cues intricately communicate with intercellular crosstalk. For instance, helminth-derived ES products (ESPs) can activate dendritic cells (DCs), leading to the development of Th2 (T helper type 2) cells and T_reg_ (regulatory T) cells. These cells help the parasite evade immune detection and ensure survival (Figure 1). Short-chain fatty acids (SCFAs) like acetate, butyrate, and propionate, which are not produced by the host but by commensal bacteria, play a role in promoting T_reg_ cells [15] (Figure 1). Hence, dysbiosis can disrupt this pathway, leading to pathogenic outcomes [16,17]. Interestingly, it is possible that helminths can also synthesise compounds [18] that promote commensal bacteria, increasing SCFA production (Figure 1) [19]. Octadecanoic acid detected in the *Nippostrongylus brasiliensis* infective larval stage (L3) helps lyse host red blood cells [5,20]. Succinic acid in the ESPs of adult *N. brasiliensis* can induce intestinal tuft cells to initiate Th2 responses and serve as an energy source during anaerobic adaptation [5,21]. Other small molecules include prostaglandins (PGs), such as PGD2 and PGE2, produced by *Brugia malayi* (filarial parasites) [22], *Onchocerca volvulus* [23], and *Schistosoma mansoni* (cercariae stage) [24]. One recently explored host–helminth interaction method uses extracellular vesicle (EV) microRNAs to influence the host immune system [25]. For example, the microRNAs secreted by *Schistosoma mansoni* (e.g., sma-microRNA-10) can manipulate the NF-κB pathway to influence the fate of host T cells. *Schistosoma* species like *S. mansoni* produce over 200 miRNAs [26] and a detailed discussion of this subject is beyond the scope of the current review.

## 3. Techniques Used for Studying GIPs

An analysis of the available literature on GIP metabolomics and lipidomics studies highlights the use of a diverse range of analytical and identification tools (Table 1). The most commonly employed techniques include liquid chromatography–mass spectrometry (LC-MS), gas chromatography–mass spectrometry (GC-MS), capillary electrophoresis–mass spectrometry (CE-MS), and nuclear magnetic resonance spectroscopy (NMR) [3]. Additionally, some studies have utilised advanced techniques such as Raman and Fourier transform infrared (FTIR) spectroscopies, atmospheric pressure matrix-assisted laser desorption/ionisation mass spectrometry imaging (AP-SMALDI MSI), ultra-high-performance liquid chromatography–mass spectrometry (UHPLC/MS), and high-resolution mass spectrometry (HRMS). LC-MS is a widely used analytical technique in global metabolite profiling. LC-MS is even better when high-resolution accurate mass (HRAM) detection is applied [27,28,29], as it can detect and identify a broader range of metabolites in terms of quantity and physicochemical properties [30]. However, we came across only one study using an HRAM detector, wherein Ferreira et al. [31] identified 20 lipids from the life cycle (eggs, miracidia, and cercariae) of *S. mansoni*. Out of 28 metabolomics studies (Table 1), the highest number of studies (8) used UHPLC/MS.

Unlike HPLC, the column used in UHPLC has a particle size of ≤2 μm, allowing more flexible and efficient separation owing to high back pressure [32]. GC-MS is the second most used technique with six studies (Table 1). For instance, the ESPs of adult *Trichuris muris* and *Nippostrongylus brasiliensis* were analysed (targeted) for their metabolites using GC-MS [21,33]. However, one of the disadvantages of using GC-MS is that, often, the derivatisation of analytes is required to enhance the resolution, detection and sensitivity [34]. Moreover, GC-MS require thermally unstable analytes [35]; more about this technique has been reviewed elsewhere [35,36,37]. Metabolomics studies using NMR are rapid and non-destructive, and no derivatisation is required, unlike GC-MS [34]. The metabolites of *Echinococcus multilocularis* (metacestode larval stage) and adult *N. brasiliensis* ESPs were analysed using proton NMR (H^1^NMR) [38,39]. NMR is also not ideal in terms of sensitivity, and it gives convoluted spectra and often more than one peak per component [34]. Recent publications by Whitman et al. [40] and Kokova and Mayboroda [41] have provided excellent reviews of GC-MS and NMR methodologies and their applications in helminths metabolomics, respectively.

Matrix-assisted laser desorption/ionisation MS imaging (MALDI-MSI) is often used in metabolomics studies [42,43]. Three studies have analysed (both targeted and untargeted) the somatic extracts of *Schistosoma mansoni* using MALDI-MSI (Table 1) [42,43,44]. One of the advantages of the MALDI technique, such as atmospheric pressure SMALDI-MSI (AP-SMALDI-MSI), over the classical LC-MS-based lipidomics method is that the spatial distribution of a wide variety of compounds can be determined immediately in tissue in a semi-quantitative manner. Kadesch et al. [44] performed an untargeted analysis of the tissue- and sex-specific lipids of adult *S. mansoni* using AP-SMALDI-MSI and identified 320 lipids (Table 1). However, some isobaric lipid species (possessing exactly or nearly the same mass), like phosphatidylethanolamines (PEs) and phosphatidylcholines (PCs), are not easily discriminated based on MSI data and must be identified using fragmentation experiments [44]. The MALDI platform is more commonly applied to analyse polymer samples [45,46], but in metabolomics, it reveals the metabolite distribution in a tissue sample [47]. Mass spectrometry with electrospray ionisation (ESI) efficiency scales in both positive and negative modes unified in a single system has enabled a comparative analysis of IE values across ionisation modes [48].

Many metabolomics/lipidomics studies prefer LC coupled with MS/ESI(+/−) since more compounds are expected to ionise in the positive mode. Moreover, the negative mode (ESI−) reduces background noise [49,50]. Lipidomics analyses of the somatic tissue extracts of *S. mansoni* [51], *Trichinella papuae* [52], and *Haemonchus contortus* [53] have applied both positive and negative ESI modes (Table 1) and identified more than 1000 lipids. However, there is a lack of proper guidelines on which ESI mode to proceed with when compounds ionise in both modes [48]. One of the main challenges in helminth metabolomics is confirming the definite source of metabolites, as it can be influenced by multiple factors, including the host, strains, sex of the helminths, and isolation timing of metabolites from the host, as well as the different technical platforms and experimental conditions applied [54]. Metabolomics and lipidomics application in helminth studies is still an emerging field of science, and methodologies, techniques, software, and bioinformatics tools are evolving.

**Table 1:** Metabolomics approaches, analytical platforms, identification databases, and software used to study the small molecules of gastrointestinal parasites.

Helminth Species and Family	Life Cycle Stage	Host	Sample Analysed	Study Approach	Metabolite Types	MSI Identification Level	Analytical Instruments/PLATFORMS Used	Databases/Software Used	Ref.
*Ancylostoma caninum* (Ancylostomatidae)	Adult	Dog	SE, ESP	Targeted	Polar metabolites and lipids	Level 1	GC-MS and LC-MS	Database: MAML Software: Agilent MassHunter (v.7); MetaboAnalyst (v.3.0)	[55]
*Ascaris suum* (Ascarididae)	L3, L4, adult	Swine	SE	Untargeted	Lipids	Level 2	UHPLC-MS/MS	Database: LipidSearch (v.4.2.23)	[56]
*Ascaris lumbricoides* (Ascarididae)	Adult	Human and swine	ESP	Targeted	Lipids	Level 1	GLC	Lipids were identified by matching retention times with standards	[57]
	Eggs, L1, L3		SE	Fingerprint	Biomarkers (pheromones/steroidal prohormones)	Level 2	HRMS	Database: Lipid MAPS; HMDB (v 3.6); METLIN Software: MetaboAnalyst (v.3.0)	[58]
*Brugia malayi* (Onchocercidae)	Adult	Dogs and wild felids	Cuticle	Targeted	Lipids	Level 1	TLC and GC	Lipids were identified by matching retention times with standards	[59]
*Dictyocaulus viviparus* (Dictyocaulidae)	Eggs, L1-L3, preadult, adult	Cattle	SE	Targeted	Lipids	Level 1	GC	Lipids were identified by matching retention times with standards Software: Chem Station B.01.03.	[60]
*Dipylidium caninum* (Dipylidiidae)	Adult	Dog	ESP	Targeted	Polar metabolites and lipids	Level 1	GC-MS	Database: MHL; KEGG; NIST library; MAML Software: MetaboAnalyst (v.4.0)	[61]
*Echinococcus multilocularis* (Taeniidae)	Larval metacestode	Fox	CS	Untargeted	Polar metabolites	Level 1	^1^H NMR	Database: HMDB Software: Chenomx NMR Suit (v.8.2); STOCSY	[62]
*Haemonchus contortus* (Trichostrongylidae)	Eggs, L3, xL3, L4, adult	Goats and sheep	SE	Untargeted	Lipids	Level 2	UHPLC-ESI(+)-MS/MS-Orbitrap	Database: LipidSearch (v.4.1.30 SPI) Software: R package (v.1.6.18)	[53]
*Hymenolepis diminuta* (Hymenolepididae)	Infective stage	Rodents (rats)	SE	Targeted	Lipids	Level 1	TLC, CC, and GLC	NA	[38]
*Necator americanus* (Ancylostomatidae)	L3	Human	SE, ESP	Untargeted	Polar metabolites	Level 1	Q-Exactive Orbitrap and MS/HPLC	Database: KEGG; MetaCyc; CTS; Lipid MAPS; PubChem; HMDB Software: IDEOM; MetaboAnalyst (v.3.0)	[63]
					Lipids	Level 2			
*Nippostrongylus brasiliensis* (Heligmonellidae)	Adult	Rodents (rats)	ESP	Targeted	Polar metabolites and lipids	Level 1	^1^H NMR	Database: GenBank; NCBI GEO Software: STAR; Chenomx NMR Suite (v.5.1)	[39]
	L3		SE, ESP	Untargeted	Polar metabolites	Level 1	Q-Exactive Orbitrap and MS/HPLC	Database: KEGG, MetaCyc; Lipid MAPS; PubChem CID; HMDB; CTS Software: IDEOM; MetaboAnalyst (v.3.0)	[21,33]
					Lipids	Level 2			
	Adult		ESP	Targeted	Polar metabolites and lipids	Level 1	GC-MS	Database: MAML; MHL; KEGG Software: Agilent MassHunter (v.7)	
	Adult		ESP	Untargeted	Polar metabolites	Level 1	UHPLC-MS	Database: HMDB; PubChem CID Software: XCMS; MetaboAnalyst (v.5.0); R package (Ropls)	[64]
			Intestinal content						
*Oesophagostomum dentatum*; *O. quadrispinulatum* (Strongylidae)	L3, L4, adult	Common livestock (goats, sheep, and swine)	SE	Untargeted	Lipids	Level 1	GC	Lipid identification: matching retention times with standards Software: MIDI software package (MIS v.3.30)	[65]
*Schistosoma mansoni* (Schistosomatidae)	Adult	Human	SE	Targeted	Lipids	Level 1	MALDI MSI (+)	Database: METLIN; Lipid MAPS Software: Uscrambler (v.9.7); Mass Frontier (v.6.0)	[42]
	Eggs, miracidia, cercariae		SE	Untargeted	Lipids	Level 2	ESI(+)-HRMS	Database: Lipid MAPS; METLIN Software: Unscrambler (v.9.7)	[31]
	Adult		SE	Untargeted	Lipids	Level 2	MALDI-MSI(+)	Database: Lipid MAPS; METLIN Software: Unscrambler (v.9.7)	[43]
	Adult		TS	Targeted	Lipids	Level 2	HPLC-MS (Sciex 4000QTRAP)	Lipids were identified by universal HPLC-MS method Software: Markerview (v.1.0)	[66]
	Eggs, cercariae, adult		SE, ESP	Targeted	Lipids	Level 2	LC-MS/MS (QTrap) (ESI−)	Software: LipidBlast; FiehnO lipid database in MS-DIAL (v2.74) Software: R package (CRAN R, v.3.3.2)	[51]
				Targeted	Lipids		GC-MS		
				Targeted	Lipids		LC-MS/MS (QToF) (ESI+)		
	Adult		SE	Untargeted	Lipids	Level 2	AP-SMALDI MSI	Database: SwissLipids; LipidMatch (v.2.0.2) Software: Lipid Data Analyzer (v.2.6.2)	[44]
*Strongyloides ratti* (Strongylidae)	L1, L3, free-living	Rodent (rats)	SE	Targeted	Lipids	Level 1	GC-MS	Lipids were identified by matching retention times with standards	[67]
*Trichuris muris* (Trichuridae)	Embryonated eggs	Rodents (mice)	SE	Untargeted	Polar metabolites	Level 1	Q-Exactive Orbitrap and MS/HPLC	Database: KEGG; MetaCyc; Lipid MAPS; PubChem CID; HMDB; CTS Software: IDEOM; MetaboAnalyst (v.3.0)	[33]
					Lipids	Level 2			
	Adult		ESP	Targeted	Polar metabolites and lipids	Level 1	GC-MS	Database: MAML; MHL; KEGG Software: Agilent MassHunter (v.7); MetaboAnalyst (v.3.0)	[21]
*Trichinella papuae* (Tricinellidae)	L1 (muscle-stage)	Swine	SE	Untargeted	Lipids	Level 2	ESI(+/−) UPLC-MS/MS	Database: Lipid MAPS; LipidBlast Software: Progenesis QI (v.2.1); QuickGO	[52]
*Toxocara canis* (Toxocaridae)	Adult	Dog	ESP	Targeted	Polar metabolites and lipids	Level 1	GC-MS and LC-MS	Database: Agilent MassHunter (v.7); MAML Software: MetaboAnalyst (v.3.0)	[54]
	Adult		SE	Untargeted	Polar metabolites and lipids	Level 1	^1^H NMR	NA	[68]

Abbreviations: Analytical techniques: AP-SMALDI MSI—atmospheric pressure (AP) matrix-assisted laser desorption/ionisation (MALDI) mass spectrometry imaging (MSI); CC—column chromatography; ESI(+/−)—electrospray ionisation positive/negative mode; GC—gas chromatography; GLC—gas liquid chromatography; ^1^H NMR—proton nuclear magnetic resonance; HPLC—high-performance liquid chromatography; HRMS—high-resolution mass spectrometry; NA—not available; LC—liquid chromatography; MS—mass spectrometry; Q-TOF—quadrupole time-of-flight; Qtrap—quadrupole ion trap; TLC—thin-layer chromatography; UHPLC—ultra-high-performance liquid chromatography. Databases: CTS—Chemical Translation Service; HMDB—Human Metabolome Database; KEGG—Kyoto Encyclopedia of Genes and Genomics; MHL—Mass Hunter Library; MAML—in-house Metabolomics Australia Metabolite Library; Matlab—matrix laboratory; MetaCyc—metabolic pathways and enzymes database; METLIN—Metabolite and Chemical Entity Database; NIST—The National Institute of Standards and Technology. Software: STAR—Spliced Transcripts Alignment to a Resonance; STOCSY—statistical total correlation spectroscopy; IDEOM—an Excel interface for analysis of LC-MS-based metabolomics data.

## 4. Metabolomics and Lipidomics Approaches and Metabolite Identification Levels

### 4.1. Approaches

**Figure 2:**
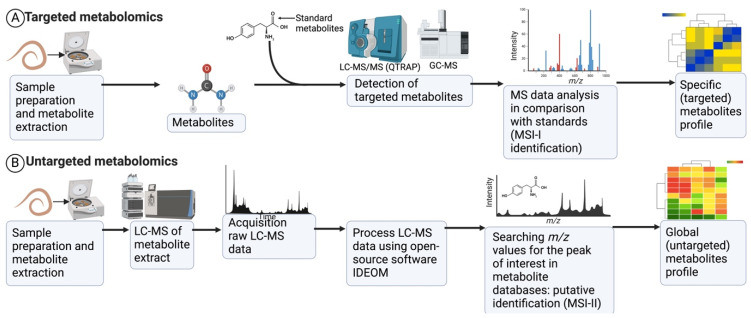
A generic LC/MS-based workflow for targeted and untargeted metabolomics studies [21,33,55]. (**A**) In targeted metabolomics, initially, targeted methods are established using standard metabolites. Method standardisation is followed by sample preparation. Samples are analysed, and the data output quantifies the metabolites for which standards are available or standard methods are established. (**B**) In an untargeted analysis, molecules are first extracted from the sample and subsequently analysed by LC-MS. Open-source software such as IDEOM is used to process the acquired LC-MS data. The putative identification of metabolites is achieved by searching the *m*/*z* values for peaks of interest in metabolite databases (Kyoto Encyclopedia of Genes and Genomes, KEGG; MetaCyc for polar metabolites; Lipid MAPS for lipids; NIST database). The untargeted approach in metabolomics yields many putative metabolites (a few hundred to thousands).

Metabolomics/lipidomics studies generally employ three methods to analyse metabolites: the untargeted method, the targeted method, and metabolic fingerprinting [69]. This review focused on the first two approaches (Figure 2). Metabolic fingerprinting (exometabolomics) examines extracellular metabolites produced in response to biotic or abiotic stresses [70,71]. Of the 28 metabolomics and lipidomics studies in Table 1, 16 used targeted methods, 11 used untargeted methods, and only 1 focused on metabolic fingerprinting [58]. Interestingly, most of these publications have been reported prominently by Wangchuk and his group from the Australian Institute of Tropical Health and Medicine, which signifies their leadership in small molecule research involving helminths. Metabolic fingerprinting was performed using either NMR, FT-IR, or MS [34]. For example, the metabolic fingerprinting of *A. lumbricoides* by Melo et al. [58] was conducted using high-resolution mass spectrometry (HRMS), in which nine biomarkers were identified from eggs and L1 and L2 larval stages. Like protein-based antigens, small-molecule biomarkers can guide the development of diagnostics to detect helminth infection. Yeshi et al. [33] analysed the metabolites of *T. muris* and *N. brasiliensis* infective stages with the highest-resolution mass spectrometry (Q-Exactive Orbitrap MS).

Of the three major approaches, the targeted approach is widely used in metabolomics studies, as it can precisely quantify known metabolites, although it has low detection limits [34]. In targeted metabolomics, methods are established using standard metabolites. Method standardisation is followed by sample preparation and metabolite extraction from somatic tissue extracts or ESPs. Samples are analysed, and the data output quantifies the metabolites for which standards are available or standard methods are established. One of the limitations of a targeted analysis is that the standards should be available in purified form for spiking or developing an in-house compound spectra library [72]. Since compound standards are limited in numbers, and many are expensive, developing an in-house standard library is often challenging. Another disadvantage is that discovering novel metabolites is difficult since the targeted approach only identifies only known metabolites.

An untargeted analysis requires complex bioinformatics or computational methods, including various AI-based algorithms, and most of the detected peaks are not identifiable. In untargeted metabolomics, metabolites/lipids are initially extracted from the sample (somatic/ESPs) and subsequently analysed by analytical instruments (e.g., LC-MS and GC-MS). LC-MS/GC-MS data are acquired in three dimensions (3D), the mass-to-charge ratio (*m*/*z*), retention time (rt), and abundance. Artificial intelligence (AI)/ML is applied at this stage to transform a large amount of complex spectral data into smaller sets of features that can be further statistically analysed. Open-source IDEOM software [33,73] is commonly used to pre-process raw data (3D) into a 2D format with aligned peak values (*m*/*z* and rt) and peak intensity/abundance. IDEOM [74] is an Excel template that provides a graphical user interface (GUI) for mzMatch and XCMS data.

Following the pre-processing stage, the putative identification of metabolites is achieved by searching the *m*/*z* values for peaks of interest in metabolite databases, which are discussed in the following sections. Other open-source algorithms used for pre-processing are MZmine 2 [75], MetAlign [76], and MS-Dial [77], but none of these algorithms are yet accepted as standard/ideal algorithms in metabolomics. A few recent studies have highlighted the presence of false positives and low-quality peaks because of poor integration in many processed metabolomics data, which may affect downstream statistical analyses [71,78].

Consequently, quality control (QC) measures have been incorporated into metabolomics studies to improve data quality and reliability. One such example is the pooling of a small volume of all samples (pooled QC), as applied in a metabolomics study of the infective stages of *N. brasiliensis* and *T. muris* [33]. Taking a pooled QC may remove false positives, but there is also the risk of some correct features being removed. Moreover, it is also challenging to discriminate between true and false positives while analysing complex samples with higher risks of contamination (>50%) [79]. Therefore, advanced AI/ML-driven algorithms for peak-picking and filtration, such as the comprehensive peak characterisation (CPC) algorithm [80], MetaClean [81], NeatMS [82], and NPFimg [83], are available, and these algorithms are discussed in depth elsewhere [9,10].

In both targeted and untargeted analyses, the types and numbers of metabolites identified depend on multiple factors, including the sample preparation, quantification level, experimental objectives and conditions applied, accuracy and precision, and number of metabolites detected [84]. A valid basis for identifying and characterising metabolites is still debated, and the consensus is continuously evolving. Shulaev has reviewed the advantages and limitations of different metabolomics approaches in detail [34].

### 4.2. Metabolite Databases and Metabolite Identification Levels

#### 4.2.1. Metabolite Databases

The metabolomics and lipidomics studies rely on various metabolite databases and spectral libraries that may contain both in silico and experimental spectra, including the Human Metabolome Database (HMDB) [85], MassBank [86], the Metabolite and Tandem MS Database (Metlin 2) [87], Global Natural Product Social Molecular Networking (GNPS) [88], and MetaCyc [89] (Table 1). Both spectral and structural databases that emerged in the field of metabolomics in 2020 have been reviewed by Misra [90]. The HMDB [91], the Metlin MS 2 [92], the in-house Metabolomics Australia Metabolite Library (MAML), the Mass Hunter Library (MHL), and the NIST library (Table 1) are the most widely used databases in GIP metabolomics studies.

The Metabolite and Tandem MS Database (Metlin MS 2) and HMDB are the primary databases used to identify polar metabolites. For example, the Metlin MS 2 database has more than 85,000 MS/MS compound spectra in positive and negative ionisation modes, constituting over 4,000,000 pieces of curated HR-MS/MS data, approximately 1% of PubChem’s compounds [87]. For non-polar metabolite/lipid identification and pathway analyses, the Lipid MAPS Structure Database is widely referred to (Table 1), as it contains about 30,000 human endogenous lipids and 12,000 plant lipids and lipid metabolism and pathways based on MS/MS spectra [93]. Popular databases such as the Kyoto Encyclopedia of Genes and Genomics (KEGG) [94] and MetaCyc [94] are used to determine metabolic pathways. Although these two databases are quite similar regarding the number of reactions occurring in pathways, MetaCyc contains a broader set of data, enabling examinations of the relationships between compounds and enzymes, the spontaneous identification of reactions, and the determination of the expected range of pathways. Their comparative features have been described elsewhere [95].

There are a few molecular databases for helminths, including WormBook (http://www.wormbook.org/, accessed on 15 June 2024), WormBase (https://wormbase.org//#012-34-5, accessed on 20 June 2024), and Wormatlas (https://www.wormatlas.org/, accessed on 23 June 2024). However, these databases primarily focus on the biological aspects, including the biochemistry, genomics, and proteomics, of the model nematode *Caenorhabditis elegans*. They do not specifically cater to GIPs. While *C. elegans* is a free-living nematode, parasitic nematodes are infectious and live inside their animal or plant host, so their metabolic pathways and metabolome compositions are expected to differ. Since there are no helminth-specific small-molecule databases, there is an urgent need for a single repository.

GIP host-specific metabolome or animal tissue-derived metabolite databases are scarce. The Animal Metabolite Database (AMDB, https://amdb.online, accessed 3 September 2024) [96] and the Livestock Metabolome Database (LMDB, https://lmdb.ca, accessed 3 September 2024) [97] are examples of existing animal metabolome databases. Unlike the HMDB, these two databases contain less metabolite information from a limited number of animal species. The AMDB contains only 168 metabolites from 50 animal species [96], while the LMDB contains slightly more metabolites, including 768 from bovine, 412 from porcine, 285 from ovine, 167 from caprine, and 109 from equine species [97].

#### 4.2.2. Metabolite Identification Levels

The metabolite identification protocol proposed by the Chemical Analysis Working Group (CAWG) was the most widely applied in all metabolomics studies of GI helminth. The CAWG established the Metabolomics Standards Initiative (MSI) in 2005, following earlier efforts to standardise metabolic reporting [98]. This working group identified four different levels of metabolite identification: MSI level 1 to MSI level 4 [99]. MSI level 1 reports metabolites whose mass and retention time match internal standards. MSI level 2 identification is putative and shows only a probable structure acquired via fragmentation data from the literature, libraries, and databases. MSI level 3 reports only putatively characterised compound classes, and MSI level 4 reports only unknown compounds [100]. For identification at MSI levels 2, 3, and 4, there is no requirement to match data with authentic standards, and, instead, mass and ion fragmentation patterns are compared to the available compound libraries or databases.

More detailed criteria for different identification confidence levels in HRMS-based metabolomics analyses can be found in Schymanski et al. [101]. Most studies, i.e., 19 out of 28, achieved the level 1 identification of metabolites (Table 1). The rest of the studies achieved only level 2 identification. Yeshi et al. [33] recently reported 55 polar metabolites through MSI level 1 identification from the infective stages of *T. muris* and *N. brasiliensis* using HRMS (Q-Exactive Orbitrap MS/HPLC). Additionally, 322 lipids were putatively (MSI level 2) identified. Wangchuk et al. [21] also identified 51 metabolites (35 polar and 16 lipids) from the adult ESPs of *T. muris* via a targeted analysis using GC-MS, of which 17 compounds were associated with various pharmacological properties. Many metabolomics studies reported putative metabolites and rarely compared their data with authentic standards due to the unavailability of these standards [100]. With the growing number of metabolomics studies and the revolution in metabolomics techniques and technologies (discussed below), greater opportunities exist for achieving higher identification rates.

## 5. Artificial Intelligence (AI)-Assisted Software and Statistical Tools for Metabolomics/Lipidomics Data Analysis

The software and statistical tools used in metabolomics studies largely depend on the mass spectrometry and spectroscopy analytical platforms used for running samples, processing raw spectra, analysing masses, and identifying molecules. The whole helminth metabolomics process can be achieved in four general steps: (i) raw data acquisition, (ii) pre-processing, (iii) post-processing, and (iv) statistical analysis and result interpretation [102]. Raw data are obtained as chromatograms (liquid chromatography), mass spectra (mass spectrometry), or NMR data. Raw data must go through pre-processing before they are further analysed.

Pre-processing usually involves alignment, binning, normalisation, and scaling processes to minimise analytical errors, reduce data points, and bring data in alignment with subsequent statistical assumptions [41]. For instance, in mass spectrometry, before pre-processing, MS data have to be converted into an open format, such as mzML, mzXML, and netCDF [102]. A recent review by Misra [90] on metabolomics tools that emerged in 2020 reported six different software tools applied in pre-processing LC-MS/MS data. The open-source cross-platforms MAVEN (latest version 2.10.17.7) [103] and MZmine 2 (v.2.14) [75] and open-source software IDEOM [74] are a few other examples that Misra did not report. Software tools such as MetaboliteDetector (v.3.3), MET-IDEA (v.2.08), metaMS (V.1.10.0), MSeasy, and SpectConnect are used with GC-MS; for additional details, refer to the review by Spicer et al. [102]. XCMS and MeltDB are applicable for pre-processing LC-MS and GC-MS data [104]. Gas chromatography–mass spectrometry is used with the NIST database (https://www.nist.gov/srd) for compound identification. Chen et al. [64] used XCMS software to process UHPLC-MS in their metabolomics studies of adult *N. brasiliensis* (Table 1). IDEOM has automated noise filtering and annotation procedures, and it can identify metabolites with high confidence levels [74]. Recent metabolomics studies on the infective stages of *T. muris, N. brasiliensis* [33], and *N. americanus* [63] have applied IDEOM software to process MS data (Table 1).

Before further statistical analysis, metabolomics/lipidomics data must go through post-processing, often called data pre-treatment [102]. At this stage, data usually undergo filtration, imputation, normalisation, centring, scaling, and transformation. According to Armitage et al. [105], up to 40% of metabolomics data may comprise missing values; thus, imputation (i.e., filling in missing values) is required. Normalisation, scaling, and transformation minimise the variations in metabolite concentrations between samples (technical variations) but not necessarily biological variations. Numerous post-processing tools, including those with R language packages and those that are web-based, are discussed in detail in Spicer et al. [102] and Misra [90].

After post-processing, metabolomics/lipidomics data (from MS, LC-MS, or NMR) are analysed statistically, and the selection of tools depends on the study design. The statistical analysis is either supervised (e.g., PLS-DA, partial least squares discriminant analysis, and OPLS-DA, orthogonal projections to latent structures discriminant analysis) or unsupervised (e.g., principal component analysis, PCA). Subsequently, various multivariate and univariate statistical tests are performed. Numerous statistical analysis software tools are powered by various programming languages (e.g., Python, R, C/C++, and Java) and web-based tools [90,102]. For example, MetaboAnalyst 6.0 (http://www.metaboanalyst.ca) [106] is a convenient free web-based statistical tool that can perform pre-processing and statistical analyses and generate results for interpretation. Metabolomics data from five different helminth species (*A. caninum*, *N. americanus*, *N. brasiliensis*, *T. canis*, and *T. muris*) were analysed using MetaboAnalyst 3.0 (Table 1). MetaboAanlyst 6.0 contains a suite of analytical tools applicable to both MS and NMR data, and it also enables enrichment and pathway analyses and advanced translational studies [102,106].

## 6. Conclusions

Among the gastrointestinal parasite (GIP) metabolomics and lipidomics studies reported so far, most studies (16 out of the 28 included) applied a targeted approach, followed by an untargeted approach (11 studies), and only 1 applied metabolic fingerprinting. One of the limitations of a targeted analysis is that the standards must be available in purified form for spiking or developing an in-house compound spectra library. GIP metabolomics data were mainly acquired with liquid chromatography–mass spectrometry (LC-MS), gas chromatography–mass spectrometry (GC-MS), capillary electrophoresis–mass spectrometry (CE-MS), and nuclear magnetic resonance spectroscopy (NMR). Very recently, advanced techniques such as Raman and Fourier transform infrared (FTIR) spectroscopies, atmospheric pressure matrix-assisted laser desorption/ionisation mass spectrometry imaging (AP-SMALDI MSI), ultra-high-performance liquid chromatography–mass spectrometry (UHPLC/MS), high-resolution mass spectrometry (HRMS), quadrupole time-of-flight (Q-TOF), and quadrupole ion trap (Qtrap) have been applied. The Metabolite and Tandem MS Database (Metlin MS 2) and HMDB are the primary databases applied to identify polar metabolites, and the Lipid MAPS databases are the primary databases applied to identify lipids.

With the emergence of many artificial intelligence (AI)/machine learning (ML)-assisted software and tools, helminth metabolomics and lipidomics have significantly advanced, particularly in the untargeted approach, enabling measurements of many metabolites, even at the trace level. Over the past decade, AI/ML advancement has accelerated significant discoveries in metabolomics and lipidomics platforms, taking the quality and reliability of data to the next level. However, “omics” technology has only recently been applied to study the small molecules produced by helminths. Even though there are limited metabolomics/lipidomics studies involving helminths, this field is gaining momentum, as many identified metabolites are associated with their immunomodulatory roles during infection or in host–parasite interactions. One of the main bottlenecks in metabolomics/lipidomics studies on GIPs is the difficulty in obtaining/collecting live worm samples from the hosts or host body. The worms must be retrieved from the host body/specific organ as quickly as possible to keep them alive. There are only limited helminth databases (e.g., WormBook and WormBase databases and a few GIP host-specific databases), but even these databases are primarily dedicated to model nematode organism, *Caenorhabditis elegans* and do not specifically cater to other GIPs. Thus, future research should focus on understudied parasitic helminths and work towards developing a single repository of GIP-specific small-molecule database.

## Data Availability

No new data were created or analysed in this study.
